# Whole Genome Sequencing of SARS-CoV-2 Strains in COVID-19 Patients From Djibouti Shows Novel Mutations and Clades Replacing Over Time

**DOI:** 10.3389/fmed.2021.737602

**Published:** 2021-09-01

**Authors:** Ikram Omar Osman, Anthony Levasseur, Ludivine Brechard, Iman Abdillahi Hassan, Idil Salah Abdillahi, Zeinab Ali Waberi, Jeremy Delerce, Marielle Bedotto, Linda Houhamdi, Pierre-Edouard Fournier, Philippe Colson, Mohamed Houmed Aboubaker, Didier Raoult, Christian A. Devaux

**Affiliations:** ^1^IHU Méditerranée Infection, Marseille, France; ^2^Aix-Marseille University, Institut de Recherche pour le Développement (IRD), Assistance Publique - Hôpitaux de Marseille (AP-HM), MEPHI, Marseille, France; ^3^Laboratoire de Diagnostic, Centre de Soins 1, Caisse Nationale de Sécurité Sociale (CNSS), Djibouti, Djibouti; ^4^Centre National de la Recherche Scientifique, Marseille, France

**Keywords:** SARS-CoV-2, UK variant, South African variant, Marseille 4 variant, COVID-19, epidemics, Djibouti

## Abstract

Since the start of COVID-19 pandemic the Republic of Djibouti, in the horn of Africa, has experienced two epidemic waves of the virus between April and August 2020 and between February and May 2021. By May 2021, COVID-19 had affected 1.18% of the Djiboutian population and caused 152 deaths. Djibouti hosts several foreign military bases which makes it a potential hot-spot for the introduction of different SARS-CoV-2 strains. We genotyped fifty three viruses that have spread during the two epidemic waves. Next, using spike sequencing of twenty-eight strains and whole genome sequencing of thirteen strains, we found that Nexstrain clades 20A and 20B with a typically European D614G substitution in the spike and a frequent P2633L substitution in nsp16 were the dominant viruses during the first epidemic wave, while the clade 20H South African variants spread during the second wave characterized by an increase in the number of severe forms of COVID-19.

## Introduction

Since the identification of severe acute respiratory syndrome coronavirus 2 (SARS-CoV-2) in China in late 2019 as the causative agent of COVID-19 (1–3), the virus has spread worldwide causing a global pandemic. Over the course of 17 months, the COVID-19 pandemic caused more than 3.69 million deaths and 171.74 million confirmed cases worldwide (4). SARS-CoV-2 genomic epidemiology has led to the detection of several mutations of the original Wuhan-Hu-1 strain of SARS-CoV-2. During the spring of 2020, a non-synonymous mutation leading to a spike protein substitution D614G became dominant in the reported sequences of SARS-CoV-2 and was found to enhance viral replication as a result of higher affinity for the ACE2 receptor (5, 6). Since the summer of 2020, the emergence of major viral variants has been observed (7–10). These variants have been found to be responsible for juxtaposed or successive epidemics in various geographic areas. Cases of re-infection with SARS-CoV-2 genotypes different from those which first infected patients, have also been documented (11). In order to track the evolution of the virus over time, many laboratories have performed virus genotyping. Laboratories equipped with whole genome sequencing capabilities have reported a large number of mutations which has increased over time. However, there are considerable disparities between countries and in some cases there is no database on circulating viruses (12).

**Figure 1:**
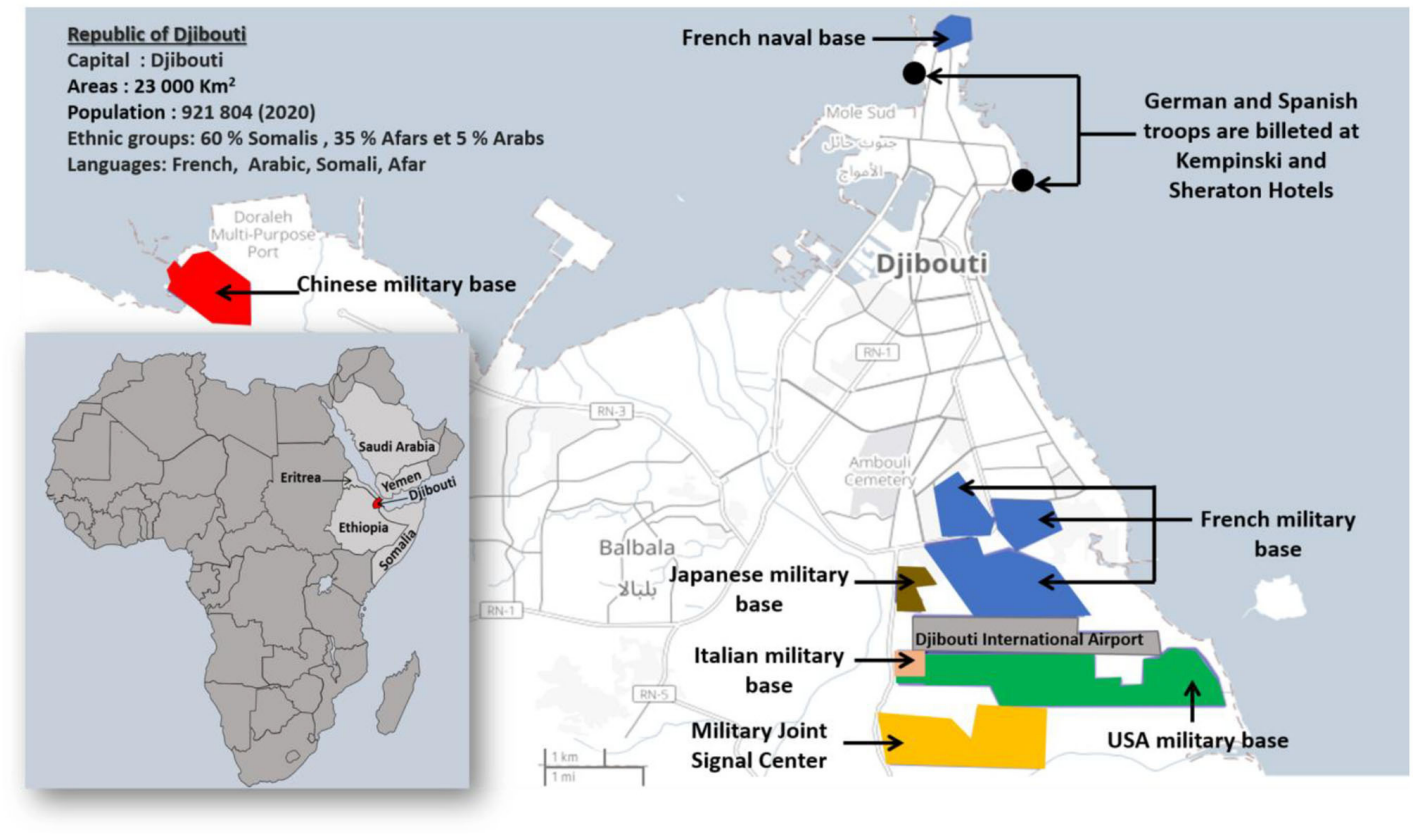
Map of Djibouti. The Republic of Djibouti is located in the horn of East Africa. In 2020, the population of the Republic of Djibouti consisted of 60% Somalis, 35% Afars, and 5% Arabs with a total of 921,804 inhabitants. The map of Dijbouti was adapted from the Open Street Map (http://umap.openstreetmap.fr/). The geographical and geopolitical situation of Djibouti is interesting because it hosts several military bases and forces from different countries including France, Italy, Germany, Spain, China, the USA, and Japan.

The Republic of Djibouti is a country in East Africa with a population of 921,804 inhabitants in 2020 composed of 60% Somalis, 35% Afars, and 5% Arabs (Figure 1). To date no SARS-CoV-2 sequence has been characterized in the Djiboutian population although cases of COVID-19 have been declared by health authorities in March 2020 (13). In response to the WHO alert on the COVID-19 pandemic, the Republic of Djibouti has rapidly established a prevention strategy based on testing, isolation, treatment and tracing of contacts of each positive case (14). Being prepared for the prevention of the epidemic risk was perhaps more essential here than elsewhere due to regional specificities. Djibouti's geopolitical situation is interesting because it hosts the military bases of many countries such as China and the USA, as well as European countries including the largest French military base in Africa. This has led to a mixing of populations which is conducive to the circulation of pathogens, including SARS-CoV-2. The first COVID-19 case in Djibouti was confirmed on 18 March 2020 in a member of the Spanish special forces (15, 16). This patient was isolated and had no interaction with the local population (17). A second COVID-19 case was reported in the same month in a member of United States Department of Defense who was working in the US military base in Djibouti (18). The first epidemic period began on 8 April and lasted until 15 August 2020 with a peak in late May early June. A second epidemic wave period began on 21 February and lasted until 18 May 2021 with a peak in late March-early April. According to the most recent (23 June 2021) information available through the Johns Hopkins Resource Center, 11,595 total confirmed cases of SARS-CoV-2 infections and 155 deaths from severe forms of COVID-19 had been reported in Djibouti, (https://coronavirus.jhu.edu/).

Our main objective was to reduce the large data gaps regarding the genomic characterization of SARS-CoV-2 circulating in Djiboutian population. Here we used mini-spike sequencing to type strains and whole genome sequencing to evaluate the diversity of SARS-CoV-2 strains that had spread in the Djiboutian population. In addition, we compared these sequences to the fifty-two SARS-CoV-2 sequences which were very recently released by the GISAID database and which corresponded to viruses that had infected foreigners working for the US Department of Defense in Djibouti.

## Materials and Methods

### Patients' Samples

Sputum and nasopharyngeal swab fluid (5–10 mL) samples from patients who had been diagnosed as positive for COVID-19 (lab-confirmed specimen by a quantitative reverse transcriptase-polymerase chain reaction, qRT-PCR) were collected in Djibouti (“Centre de soins 1 de la Caisse Nationale de Sécurité Sociale,” Social Security Care center number 1 of the Republic of Djibouti). The bank of samples (1,861 samples collected, including 407 SARS-CoV-2 positive samples) was kept in Djibouti until use and only fifty three frozen samples from individual with a cycle threshold (Ct) value below 25 [see column “Ct(Dji)” in Table 1] corresponding to a high viral load, were transferred in biohazard containers (Aramex company) to the IHU Méditerranée Infection institute (Marseille, France). Among these fifty three samples representative of the epidemiological situation, twenty-five were collected during the period from May 2020 to August 2020; three were collected during the inter-waves period (October 2020 to January 2021); and twenty-five were collected during the second epidemic wave in March 2021. Before being processed, each transferred sample was retested using a qRT-PCR performed with specific primers for the E gene of SARS-CoV-2, to confirm the presence of virus in sample after transport [see column “Ct(IHU)” in Table 1]. All samples received at the IHU showed CT values below 25, with the exception of sample number 00100 found with a CT of 32.4. According to our diagnostic criteria, this sample remained positive since the qRT-PCR are considered positive when the Ct value is below 35, as previously reported (19). Only samples that meet a Ct < 25 were transferred to the IHU Marseille genomic sequencing platform for mini-spike sequencing and/or whole genome sequencing.

**Table 1:** Typing of SARS-CoV-2 strains circulating in Djibouti from May 2020 to March 2021.

	**SARS-CoV-2 samples**			**SARS-CoV-2 spike typing using specific probes**			**Mini-spike sequencing**	
**Sample^**a**^**	**Sampling date (Djibouti)**	**Ct (Dji)/Ct (IHU)^**b**^**	**SARS-CoV-2 E gene^**c**^**	**Marseille 4 Variant**	**UK Variant**	**South African Variant**	**Mutation**	**Cluster**
			**qRT-PCR**	**A23403G (20A).EU2 (N477S)^**d**^**	**20I/501Y.V1 (N501Y)**	**20H/501Y.V2 (E484K, N501Y)**		
**First epidemic wave**								
B	02 May 2020	23.3/22.3	Positive	Negative	Negative	Negative	Undetermined	—
00030	21 May 2020	20.7/17.5	Positive	Negative	Negative	Negative	A23403G (20A)/C21365T mut.	3
00034	21 May 2020	22.0/19.4	Positive	Negative	Negative	Negative	A23403G (20A) (Wuhan-Hu-1-like)	1
00057	22 May 2020	19.2/17.0	Positive	Negative	Negative	Negative	A23403G (20A)/C21365T mut.	3
00062	22 May 2020	22.0/17.1	Positive	Negative	Negative	Negative	A23403G (20A)/C21365T mut.	3
00094	26 May 2020	22.0/18.3	Positive	Negative	Negative	Negative	A23403G (20A)/C21365T mut.	3
00095	26 May 2020	18.9/17.3	Positive	Negative	Negative	Negative	A23403G (20A)/C21365T mut.	3
00099	26 May 2020	21.9/22.4	Positive	Negative	Negative	Negative	A23403G (20A)/C21365T mut.	3
00100	26 May 2020	22.9/32.4	Positive	Negative	Negative	Negative	Undetermined	–
00103	26 May 2020	22.5/19.9	Positive	Negative	Negative	Negative	A23403G(20A)/C21365T,G21624C	6
00104	26 May 2020	21.8/20.3	Positive	Negative	Negative	Negative	Undetermined	–
00115	26 May 2020	21.2/15.6	Positive	Negative	Negative	Negative	20B (Wuhan-Hu-1-like)	1
00138	27 May 2020	22.9/19.4	Positive	Negative	Negative	Negative	A23403G (20A)/C21365T mut.	3
00151	27 May 2020	20.5/21.4	Positive	Negative	Negative	Negative	A23403G (20A) (Wuhan-Hu-1-like)	1
00152	27 May 2020	19.3/18.2	Positive	Negative	Negative	Negative	A23403G (20A)/C21365T mut.	3
00173	28 May 2020	22.1/18.5	Positive	Negative	Negative	Negative	Undetermined	–
00182	29 May 2020	18.1/15.9	Positive	Negative	Negative	Negative	A23403G (20A)/C21365T mut.	3
00187	29 May 2020	21.2/16.9	Positive	Negative	Negative	Negative	A23403G (20A)/C21365T mut.	3
00206	31 May 2020	22.9/16.0	Positive	Negative	Negative	Negative	A23403G (20A)/G22093C mut.	2
00326	14 June 2020	22.4/20.4	Positive	Negative	Negative	Negative	A23403G (20A)/C21365T,C23191T	5
00388	07 July 2020	20.0/16.6	Positive	Negative	Negative	Negative	A23403G (20A)/C21365T,C21789T	4
00390	07 July 2020	20.2/17.1	Positive	Negative	Negative	Negative	A23403G (20A)/C21365T,C21789T	4
00391	07 July 2020	20.0/17.3	Positive	Negative	Negative	Negative	A23403G (20A)/C21365T,C21789T	4
00420	03 August 2020	20.2/17.7	Positive	Negative	Negative	Negative	A23403G (20A) (Wuhan-Hu-1-like)	1
00498	20 August 2020	22.2/18.4	Positive	Negative	Negative	Negative	Undetermined	–
**Inter-epidemic wave**								
00666	21 October 2020	21.6/19.5	Positive	Negative	Negative	Negative	A23403G (20A) (Wuhan-Hu-1-like)	1
00700	22 November 2020	25.2/23.6	Positive	Negative	Negative	Negative	A23403G (20A)/C21365T mut.	3
00730	28 January 2021	25,2/23,6	Positive	Negative	Negative	Negative	A23403G (20A) (Wuhan-Hu-1-like)	1
**Second epidemic wave**								
804	23 March 2021	24.4/19.4	Positive	Negative	Negative	Positive	ND^e^ (S. African variant-like)	ND
805	23 March 2021	23.1/17.5	Positive	Negative	Negative	Positive	ND^e^ (S. African variant-like)	ND
807	23 March 2021	24.1/16.7	Positive	Negative	Negative	Positive	ND^e^ (S. African variant-like)	ND
809	24 March 2021	22.9/16.8	Positive	Negative	Negative	Positive	ND^e^ (S. African variant-like)	ND
836	24 March 2021	24.4/17.2	Positive	Negative	Negative	Positive	ND^e^ (S. African variant-like)	ND
890	24 March 2021	24.1/18.1	Positive	Negative	Negative	Positive	ND^e^ (S. African variant-like)	ND
896	24 March 2021	24.1/14.2	Positive	Negative	Negative	Positive	ND^e^ (S. African variant-like)	ND
952	26 March 2021	21.6/15.9	Positive	Negative	Negative	Positive	ND^e^ (S. African variant-like)	ND
957	26 March 2021	24.7/19.2	Positive	Negative	Negative	Positive	ND^e^ (S. African variant-like)	ND
1003	28 March 2021	24.4/22.5	Positive	Negative	Negative	Positive	ND^e^ (S. African variant-like)	ND
1007	28 March 2021	24.7/20.7	Positive	Negative	Negative	Positive	ND^e^ (S. African variant-like)	ND
1010	28 March 2021	16.3/14.9	Positive	Negative	Negative	Positive	ND^e^ (S. African variant-like)	ND
1019	28 March 2021	24.3/20.3	Positive	Negative	Negative	Positive	ND^e^ (S. African variant-like)	ND
1023	28 March 2021	20.7/17.0	Positive	Negative	Negative	Positive	ND^e^ (S. African variant-like)	ND
1029	28 March 2021	23.9/18.8	Positive	Negative	Negative	Positive	ND^e^ (S. African variant-like)	ND
1053	28 March 2021	23.0/16.7	Positive	Negative	Negative	Positive	ND^e^ (S. African variant-like)	ND
1088	29 March 2021	19.1/15.9	Positive	Negative	Negative	Positive	ND^e^ (S. African variant-like)	ND
1089	29 March 2021	22.4/19.0	Positive	Negative	Negative	Positive	ND^e^ (S. African variant-like)	ND
1094	29 March 2021	23.2/17.1	Positive	Negative	Negative	Positive	ND^e^ (S. African variant-like)	ND
1095	29 March 2021	24.8/19.5	Positive	Negative	Negative	Positive	ND^e^ (S. African variant-like)	ND
1181	30 March 2021	24.9/19.2	Positive	Negative	Negative	Positive	ND^e^ (S. African variant-like)	ND
1229	31 March 2021	23.3/16.2	Positive	Negative	Negative	Positive	ND^e^ (S. African variant-like)	ND
1271	31 March 2021	24.3/16.4	Positive	Negative	Negative	Positive	ND^e^ (S. African variant-like)	ND
1276	31 March 2021	21.8/17.6	Positive	Negative	Negative	Positive	ND^e^ (S. African variant-like)	ND
1283	31 March 2021	24.7/16.9	Positive	Negative	Positive	Negative	ND^e^ (UK variant-like)	ND

a *Deidentified sample; Sputum and Nasopharyngeal swab fluid samples from patients diagnosed positive for COVID-19 (lab-confirmed specimen*.

b *Ct (Dji) = > Cycle threshold measured in Djibouti before transport; Ct (IHU) = > cycle threshold measured at IHU Méditerranée Infection, Marseille after transport*.

c *E gene sequence from Wuhan HU1 strain*.

d *PCR amplification using specific probes for the Marseille 2 variant strain were also performed but turn to be negative for all samples tested (not shown*.

e *ND, Not done (the mini-spike sequencing was not done on samples already typed using the specific probes for variants*.

### SARS-CoV-2 Variant Typing and Mini-Spike Sequencing

Several specific qRT-PCR systems with primers and fluorescent dye-tagged probes (see Supplementary Table 1) that can confirm the presence of SARS-CoV-2 in samples (a pan-genomic SARS-CoV-2 probe that hybridizes with the E gene; size of the amplification fragment: 113 bp), and probes which can discriminate between the Marseille 4 variant (primers in the ORF1; size of the amplification fragment: 114 bp), UK variant (Nextstrain clade 20I; primers in the N gene; size of the amplification fragment: 110 bp), and South African variant (Nextstrain clade 20H; primers in nsp2; size of the amplification fragment: 119 bp) strains, were used for the rapid identification of variants.

Regarding the samples which were found positive for the SARS-CoV-2 E gene and negative for the three variants, a mini-spike sequencing strategy was performed as follows. the sequences of the spike gene 5'-region (nucleotides 1-1,854 in reference to NC_045512.2), named mini-spike, were obtained by next-generation sequencing (NGS) following an in-house protocol that enables the PCR amplification of a fragment between position 21,296 (in the ORF1b) and position 23,424 (in the spike gene) of the viral genome (primers: 5′-GCAAACCACGCGAACAAATA-3′; 5′-GGGACTTCTGTGCAGTTAAC-3′; size of the amplification fragment: 2,128 bp). Sequences were analyzed using Nextclade (https://clades.nextstrain.org/) (20) and an in-house Python script.

### SARS-CoV-2 Whole Genome Sequencing

A few samples were further characterized by next-generation sequencing (NGS) using the Nanopore sequencing (Oxford Nanopore Technologies, Cambridge, UK), as previously described (21). Regarding the eight samples from the first wave characterized by NGS, they were selected to be representative of the different SARS-CoV-2 clusters found using the mini-spike sequencing (e.g., the Djiboutian sample 103 from May 2020 in Table 1 corresponds to sample ID Djibouti CNSS00103-IHU1031243436/2020 in Table 2). Regarding the five samples from the second wave, we have chosen to better characterize four South African variants (e.g., the Djiboutian sample 1095 from March 2021 in Table 1 corresponds to sample ID Djibouti CNSS001095-IHU1031441326/2021 in Table 2) and the only UK variant discovered in this series of samples. The consensus sequences were generated with a minimum coverage of 100 reads. Genome consensus sequences were generated through mapping on the SARS-CoV-2 genome using the sequence from the Wuhan-Hu-1 reference strain (GenBank accession no. NC_045512.2) with the Minimap2 software using short genomic paired-end reads as pre-set (22). All the new SARS-CoV-2 sequences reported herein have been placed in the open access GISAID database (Accession ID: EPI_ISL_2820509; EPI_ISL_2820514; EPI_ISL_2820756; EPI_ISL_2820520; EPI_ISL_2820757; EPI_ISL_2820547; EPI_ISL_2820621; EPI_ISL_2820626; EPI_ISL_2820687; EPI_ISL_2820701; EPI_ISL_2820704; EPI_ISL_2820705; EPI_ISL_2820706).

**Table 2:** Genotyping of SARS-CoV-2 strains circulating in Djibouti from May 2020 to March 2021.

	**Mutations**					**GISAID accession ID**
**Samples ID**	**Spike typing**	**Cluster**	**Total of mutation**	**Total amino acid substitutions**	**Total amino acid deletion**	
Djibouti/CNSS00103-IHU1031243436/2020	A23403G (20A)/C21365T,G21624C	6	11	6	0	EPI_ISL_2820509
Djibouti/CNSS00115-IHU1031243441/2020	A23403G (20A) (Wuhan-Hu-1-like)	1	10	6	0	EPI_ISL_2820514
Djibouti/CNSS00182- IHU1031243454/2020	A23403G (20A)/C21365T mut.	3	11	7	5	EPI_ISL_2820756
Djibouti/CNSS00187-IHU1031243455/2020	A23403G (20A)/C21365T mut.	3	10	4	0	EPI_ISL_2820520
Djibouti/CNSS00206-IHU1031243458/2020	A23403G (20A)/G22093C mut.	2	11	7	0	EPI_ISL_2820757
Djibouti/CNSS00326-IHU1031243462/2020	A23403G (20A)/C21365T,C23191T	5	21	11	0	EPI_ISL_2820547
Djibouti/CNSS00388- IHU1031243469/2020	A23403G (20A)/C21365T,C21789T	4	9	8	0	EPI_ISL_2820621
Djibouti/CNSS00390-IHU1031243473/2020	A23403G (20A)/C21365T,C21789T	4	8	7	0	EPI_ISL_2820626
Djibouti/CNSS1095-IHU1031441326/2021	South African variant-like	ND	23	15	6	EPI_ISL_2820687
Djibouti/CNSS1088-IHU1031441332/2021	South African variant-like	ND	26	20	6	EPI_ISL_2820701
Djibouti/CNSS1094-IHU1031441337/2021	South African variant-like	ND	30	22	6	EPI_ISL_2820704
Djibouti/CNSS1053-IHU1031441340/2021	South African variant-like	ND	28	20	6	EPI_ISL_2820705
Djibouti/CNSS1283-IHU1031454612/2021	UK variant-like	ND	35	25	6	EPI_ISL_2820706

### SARS-CoV-2 Phylogenetic Tree

Phylogenetic reconstruction was performed using the IQ-TREE software with the GTR Model and 1,000 ultrafast bootstrap repetitions after aligning genomes using MAFFT v.7 (23); the tree was visualized with the Interactive Tree Of Life (iTOL)-v6 software, as previously described (21).

## Results

### Typing of SARS-CoV-2 Strains Spreading in Djibouti

**Figure 2:**
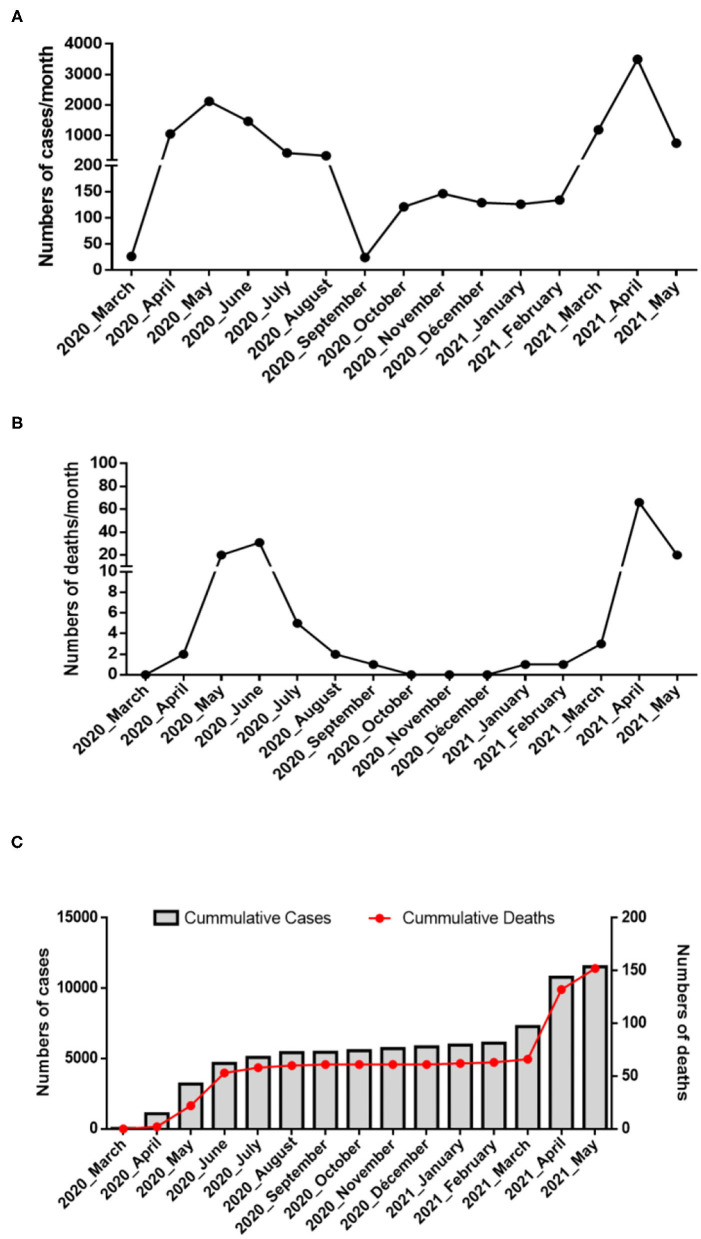
Epidemiology of COVID-19 in Djibouti. Summary of the COVID-19 outbreaks in the Republic of Djibouti. **(A)** Epidemiological data regarding the COVID-19 episodes compiled from information provided by the WHO from March 2020 to May 2021 (https://www.who.int/emergencies/diseases/novel-coronavirus-2019/situation-reports/). Three periods (first wave, inter-wave, second wave) were provisionally defined during which samples were collected for the genetic analysis of the SARS-CoV-2 strains. **(B)** Number of deaths over time. **(C)** Cumulative number of COVID-19 cases and SARS-CoV-2-associated deaths in Djibouti.

**Figure 3:**
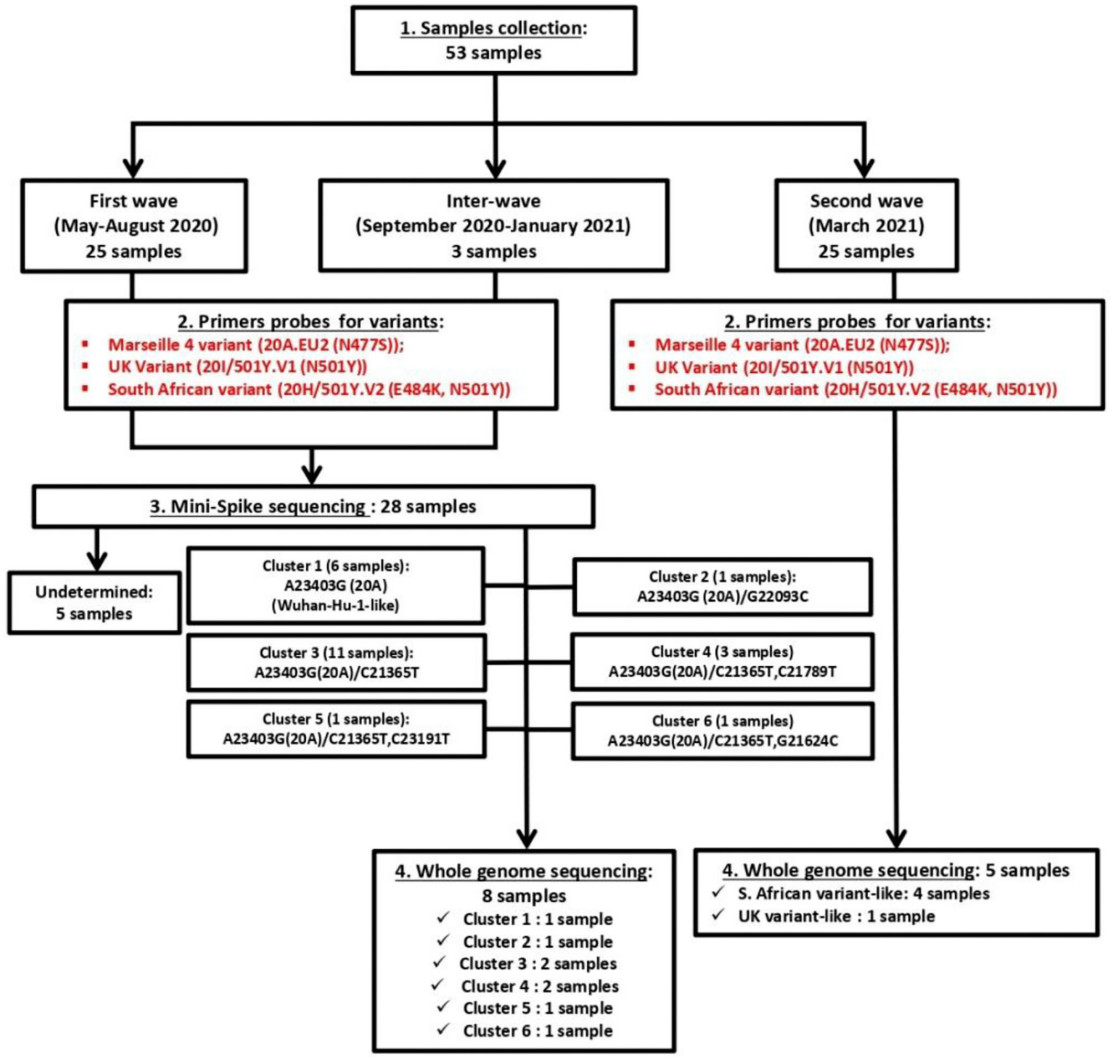
Chart of management of the fifty-three SARS-CoV-2 samples for typing and whole genome sequencing.

As summarized in Figure 2 (upper panel), the Republic of Djibouti has experienced two epidemic waves of COVID-19. The first wave occurred between April and August 2020, and the second occurred between February and May 2021. The cases fatality rate was lower during the first wave (number of cases: 5,414; number of deaths: 60; fatality rate: 1.10%) than during the second wave (number of cases: 5,560; number of deaths: 90; fatality rate: 1.61%), (Figure 2 middle panel), suggesting the circulation of more deadly viruses during the second wave. The cumulative number of confirmed severe COVID-19 cases leading to patients' deaths was 152 from March 2020 to May 2021 (Figure 2, lower panel). As shown in the chart (Figure 3), all samples were tested for SARS-CoV-2 using a PCR-detection of the viral E gene, followed by screening using specific probes that discriminate between different SARS-CoV-2 variants (routinely used at the IHU for the identification of SARS-CoV-2 variants). This fast screening strategy was successful for the typing of samples collected in March 2021 in Djibouti but failed for samples collected during the first epidemic wave and the inter-wave period because the strains circulating in 2020 had not yet accumulated the characteristic mutations of the variants. In order to successfully type these SARS-CoV-2, the samples were submitted for mini-spike sequencing. For twenty-three samples the mini-spike sequencing (fragment 21296–23424) was successful, but for the five remaining strains it was not interpretable due to poor data quality. We found (Table 1) that during the first epidemic wave, all viruses derived from the Wuhan-Hu-1 reference strain but carried the non-synonymous A23403G mutation, leading to the substitution D614G in the spike. Six viruses showed only this mutation in the mini-spike region, while most other viruses (16 strains) carried an additional non-synoymous mutation C21365T in the ORF1b, leading to a P2633L substitution in the pp1ab precursor of nsp16 methyltransferase. Additional non-synonymous mutations were found in the mini-spike of some viruses, including G21624C (leading to a substitution R21T), C21789T (leading to a substitution T76I), and G22093C (leading to a substitution M177I), all located in the spike N-terminal domain.

According to the mini-spike sequences, all SARS-CoV-2 circulating during the first epidemic wave derive from the 20A clade that evolves into two groups: one being clade 20B that comprises the ancestor viruses at the origin of the group 1 mutant virus carrying the non-synonymous mutation C18495T (leading to P1675L substitution in the ORF1b) and the synonymous mutation C19836T, and the other group comprising viruses carrying either the non-synonymous C21365T mutation compared to the 20A consensus sequence (cluster 6) or both mutations C21365T and C21789T (cluster 4). Regarding the viruses circulating during the inter-wave period, they belong to clade 20A with one virus which carried the non-synonymous mutation C21365T in the ORF1b. A completely different pattern was found for SARS-CoV-2 circulating during the second epidemic wave; one virus was identified as a UK variant (20I/501Y.V1) of SARS-CoV-2 while the large majority of the strains (24 of 25) were identified as South African variants (20H/501Y.V2).

### Whole Genome Sequencing of SARS-CoV-2 Spreading in Djibouti

Once the SARS-CoV-2 spike gene typing was performed, it became interesting to study the genetic polymorphism of these viruses using whole genome sequencing. To achieve this goal, eight SARS-CoV-2 circulating in Djibouti during the first epidemic wave (chosen on the basis of their polymorphism in the spike gene) and five SARS-CoV-2 circulating during the second epidemic wave (four South African variants and one UK variant) were submitted to whole genome sequencing (Table 2).

**Figure 4:**
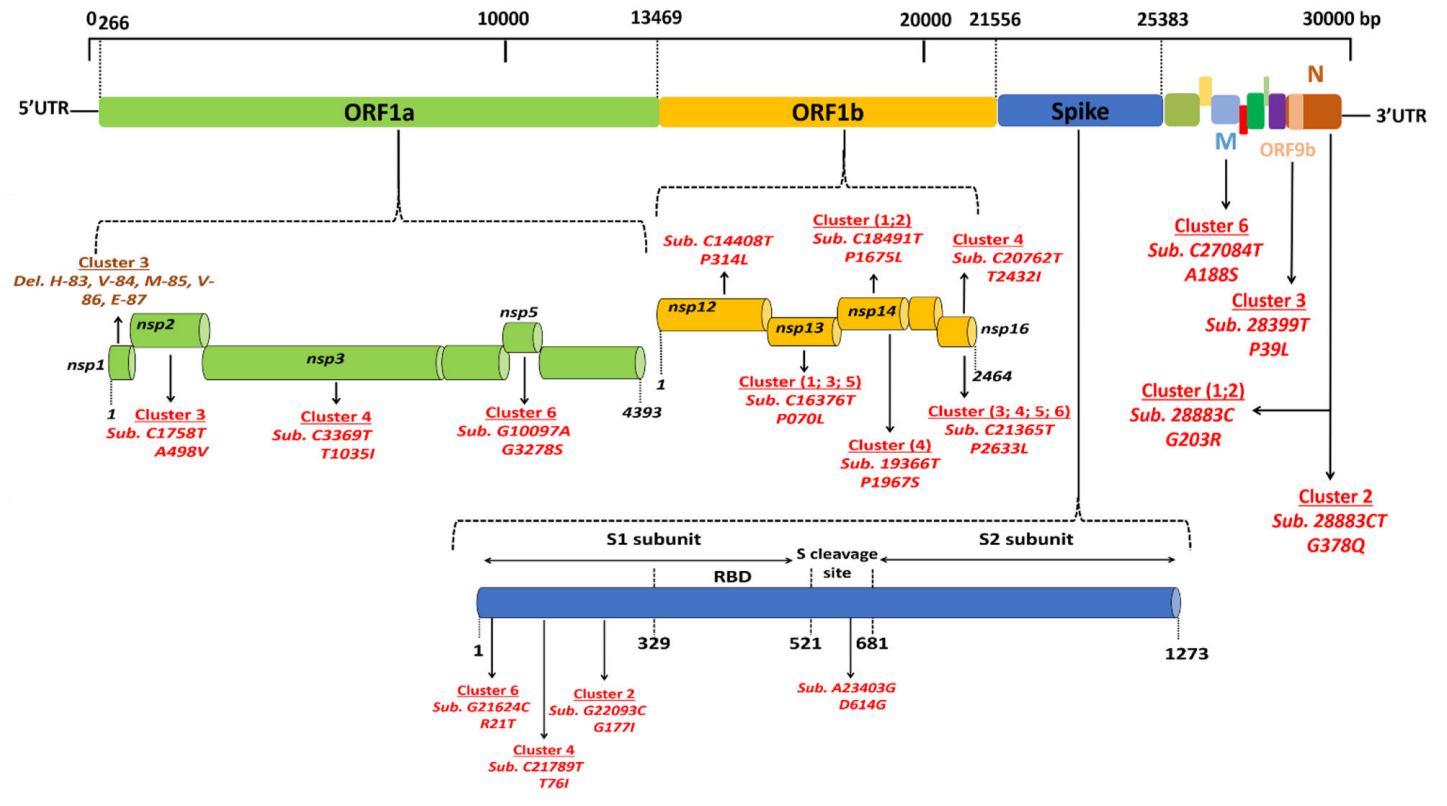
Location of genomic mutations and deletions on the complete nucleotide sequence from different isolates of SARS-CoV-2. Viruses circulating during the first epidemic wave derive from the 19A clade that evolves into two clades: 20A, including viruses from clusters 3, 4, 5, and 6, and 20B, including viruses from clusters 1 and 2. Del means deletion; Sub means substitution.

**Figure 5:**
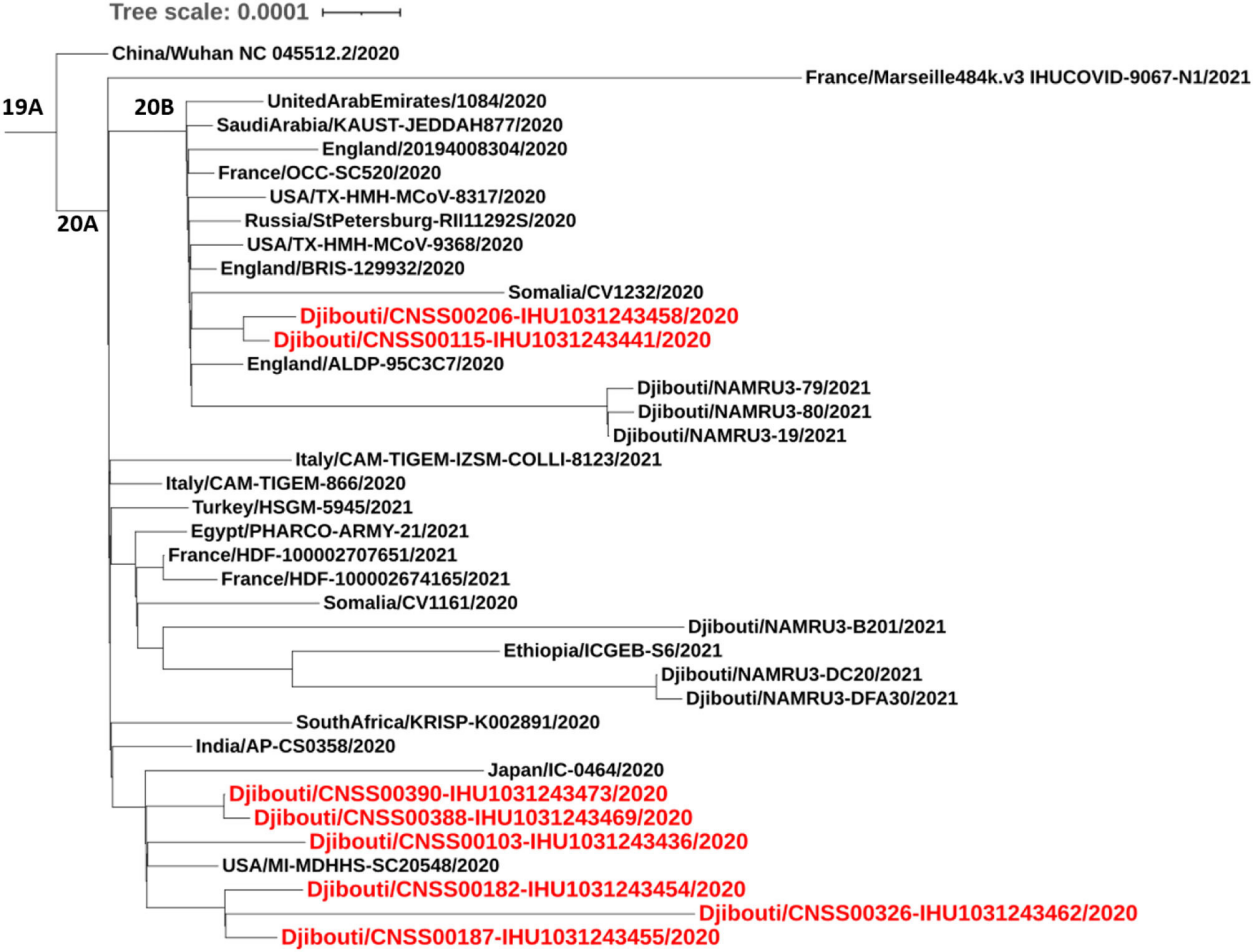
Phylogenetic tree (obtained using the IQ-TREE software with the GTR Model and 1,000 ultrafast bootstrap 84 repetitions), including height SARS-CoV-2 strains from the first COVID-19 wave in Djibouti compared to 23 SARS-CoV-2 sequences (clades 19B, 20A, 20B), available through GISAID.

Regarding viruses from the first epidemic wave, the total number of mutations along the genomes compared to the Wuhan-Hu-1 reference genome varied from eight mutations (strain code Djibouti/CNSS00390-IHU1031243473/2020) up to 21 mutations (strain code Djibouti/CNSS00326-IHU1031243462/2020). These mutations lead to a number of amino acid substitutions varying from 4 to 11 (Table 2). In addition the deletion of 5 amino acids was observed for one virus. The distribution of these mutations on the genome of SARS-CoV-2 is illustrated in Figure 4. Four substitutions affect the spike while the others are distributed in the non-structural proteins (nsp2, nsp3, nsp5, nsp12, nsp13, nsp14, nsp16), M (membrane), ORF9b, and N (nucleocapsid) proteins. As shown in Figure 5, when compared to other sequences (the closest sequences selected via blast) available through the Global Initiative on Sharing Avian Influenza Data (GISAID), the 20A SARS-CoV-2 genomes of the first epidemic wave are genetically related to genomes obtained in Japan (GISAID ID: Japan/TC-0464/2020) and in the USA (GISAID ID: USA/MI-MDHHS-SC20548/2020) while the 20B genomes are related to genomes from Somalia (Somalia/CV1232/2020) and England (England/ALDP-95C3C7/2020) characterized during the same period.

**Figure 6:**
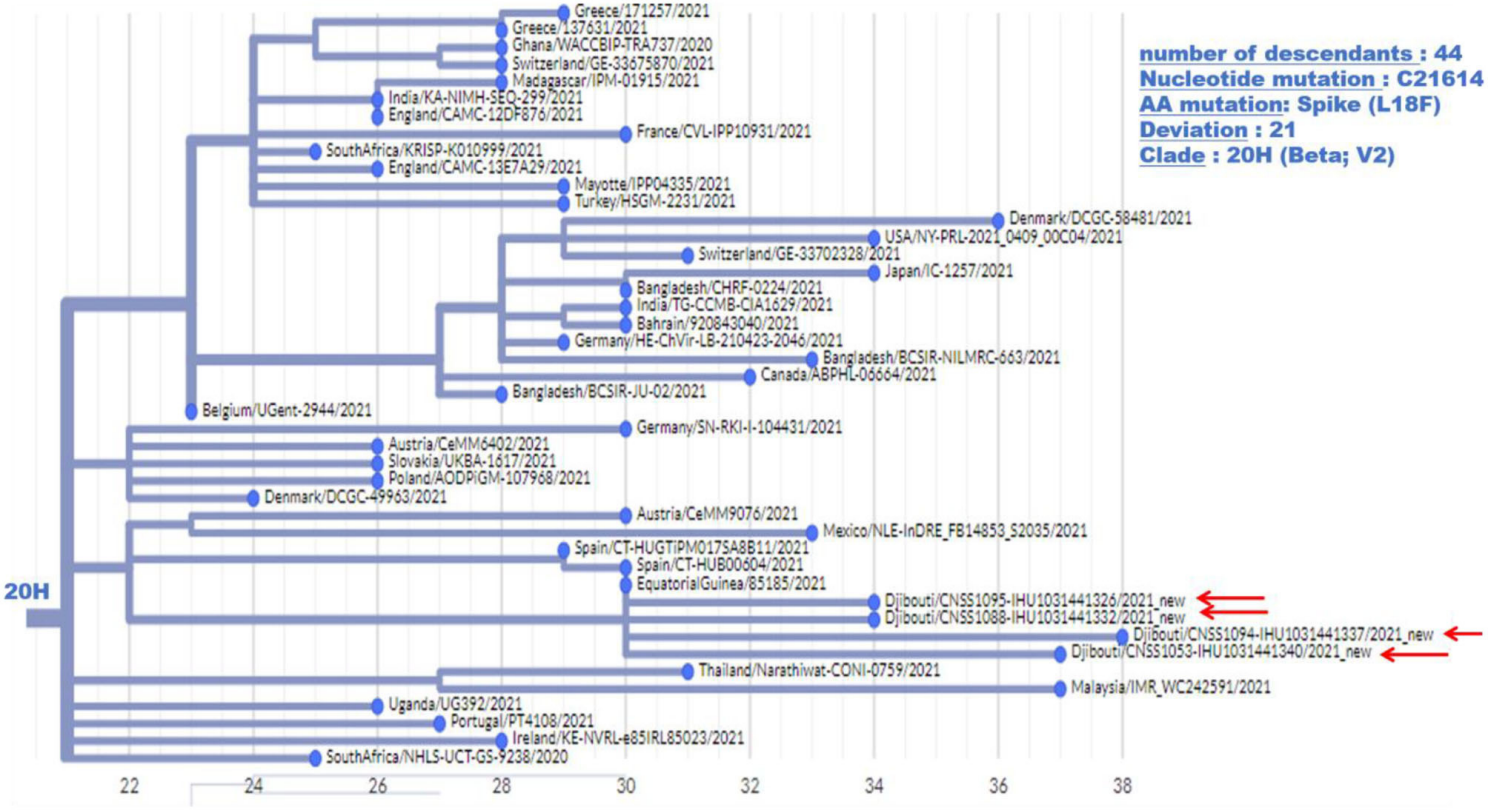
Phylogenetic tree (obtained using the Nexcladevv0.14.4 software (https://clades.nextstrain.org/), including four South African variants of SARS-CoV-2 strains from the second COVID-19 wave in Djibouti compared to forty SARS-CoV-2 sequences (clade 20H) available through GISAID and automatically selected using Nextclade. The scale at the bottom of the figure indicates the number of mutations (deviation) compared to the sequence of the reference strain (Wuhan-Hu-1 reference strain; GenBank accession no. NC_045512.2).

**Figure 7:**
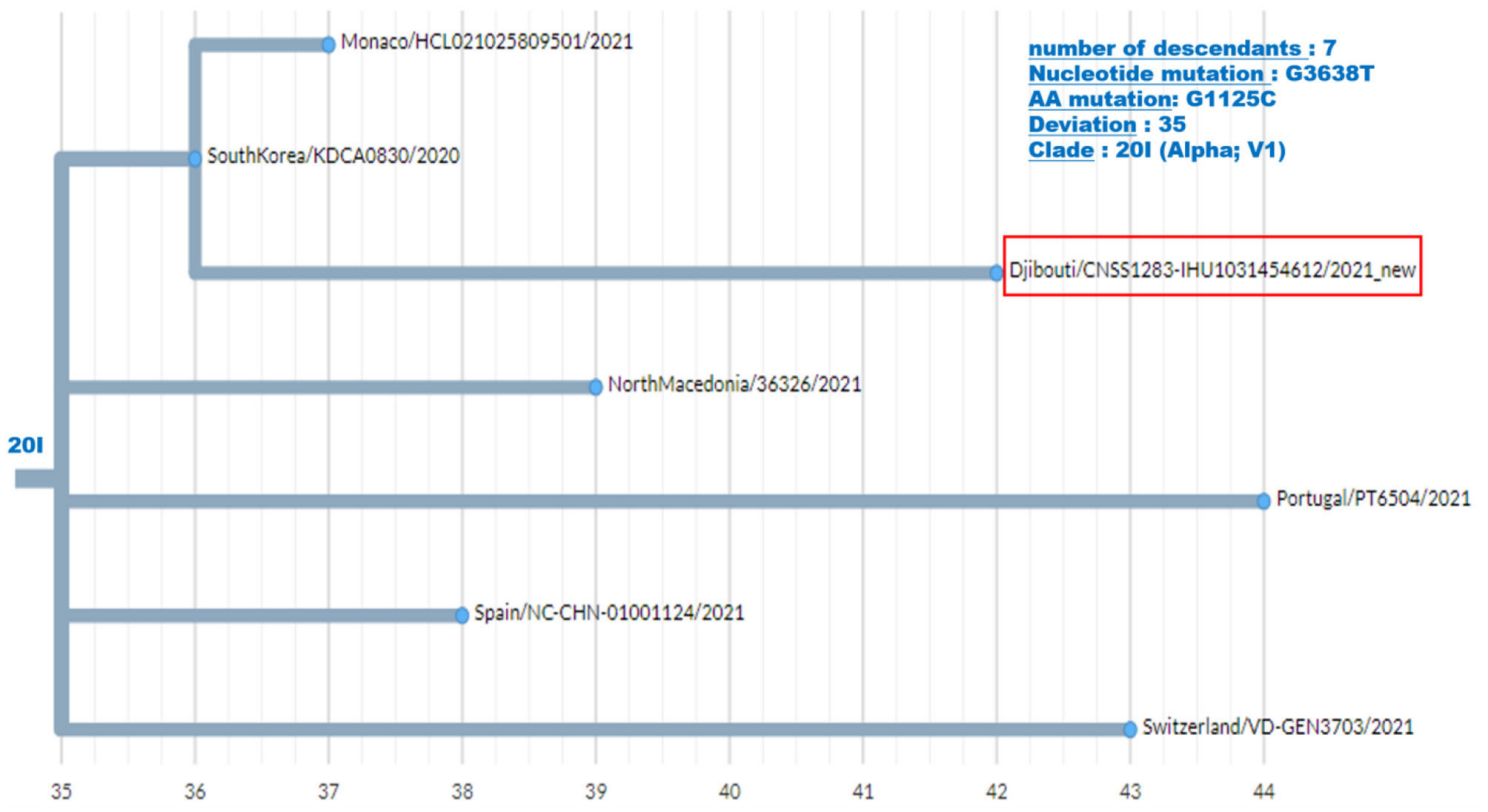
Phylogenetic tree (obtained using the Nexcladevv0.14.4 software (https://clades.nextstrain.org/), including the UK variant of SARS-CoV-2 strain from the second COVID-19 wave in Djibouti compared to six SARS-CoV-2 sequences (clade 20I) available through GISAID. The scale at the bottom of the figure indicates the number of mutations (deviation) compared to the sequence of the reference strain (Wuhan-Hu-1 reference strain; GenBank accession no. NC_045512.2).

Regarding the South African SARS-CoV-2 variant from the second epidemic wave, the four genomes obtained by NGS showed between 23 and 30 mutations compared to the Wuhan-Hu-1 genome. Most of these mutations were non-synonymous, leading to between 15 and up to 22 amino acid substitutions and five amino acid deletions (Table 2). These four viruses are genetically related to one another and belong to clade 20H. They were found to be closely related to viruses circulating in Spain (Spain/CT-HUB00604/2021) and Equatorial Guinea (Equatorial Guinea/85185/2021) circulating during the same period of 2021 (Figure 6). Regarding the only UK variant (Djibouti/CNSS1283-IHU1031454612/2021) found during the second epidemic wave, it shows 35 mutations compared to the Wuhan-Hu-1 strain, leading to 25 amino acid substitutions and six amino acid deletions (Figure 7). This genome belong to clade 20I and was genetically related to a genome circulating the previous year in South Korea (South Korea/KDCA830/2020).

## Discussion

Like what has been observed in the rest of the world, the Republic of Djibouti was confronted in 2020 with an epidemic of SARS-CoV-2 dominated by Wuhan-HU-1-like viruses. This first epidemic wave was followed in 2021 by the emergence of SARS-CoV-2 variant viruses belonging mainly to the South African lineage known to cause more severe forms of COVID-19.

When SARS-CoV-2 started to spread worldwide, the viruses that circulated were closely related to the Wuhan-Hu-1 strain, with only a few mutations. However, a D614G substitution in the spike has emerged with an increasing prevalence of this variant during the COVID-19 pandemic (24). Patients infected with this virus shed more viral nucleic acid compared with those infected with the wild-type (D614) Wuhan-Hu-1 strain and showed significantly higher infectious titers *in vitro* as a consequence of higher affinity for ACE2 (6). During the summer of 2020, the dynamic tracking of the SARS-CoV-2 mutants in the global pandemic highlighted a rapid evolution of the virus with an increase in the frequency of mutations and an epidemic pattern characterized by the generation of new variants including the Marseille 4 variant Nextclade 20A.EU2 (7), the UK variant 20I/501Y.V1 (8), the South African variant 20H/501Y.V2 (9), the Brazilian variant 484K.V2 (25), and others (10). These variants can be associated with different clinical features of COVID-19 and increased frequency of death (26, 27).

Despite the restrictive measures on international transport and the lockdown measures applied in most countries, the variants continued to spread from one geographic region to another (28). Although Africa is considered as the most vulnerable continent, it was the last to be impacted by the pandemic (29, 30). The SARS-CoV-2 was reported to have spread to Africa in February 2020, with the earliest diagnosed cases being detected in Egypt and Nigeria. The virus introduction in Africa was considered to be mainly of American and European origins rather than China. In partnership with the WHO, the Africa Task Force established a strategy to fight COVID-19 and 43 African countries were able to test for SARS-CoV-2, although test kits were in short supply. On 11 March 2020, the WHO declared COVID-19 a global pandemic. By 31 March 2020, 5,389 cases of COVID-19 had been reported in Africa (31). By 18 April 2020, the African CDC had reported 19,895 confirmed cases, including 1,017 deaths from 52 African countries. Compared to the global 7,700 genome sequences of SARS-CoV-2 available, the African continent had reported only 90 genome sequences from five countries (29). By the end of April 2020, confirmed cases were found in most African countries, with Djibouti ranking in the top seven of the most affected countries (Egypt: *n* = 3,490; South Africa: *n* = 3,465; Morocco: *n* = 3,209; Algeria: *n* = 2,811; Cameroon: *n* = 1,163; Ghana: *n* = 1,042; Djibouti: *n* = 945) (32). Despite a weak health care system and a large immune-compromised population linked to the high prevalence of malaria, HIV/AIDS, tuberculosis, and malnutrition, the progression of COVID-19 in Africa remained well-below the predictions forecasting a disaster on the continent (30). However, the second wave of COVID-19 appeared to be more aggressive, with more cases and higher fatality rates. By 31 December 2020, African countries had reported 2,763,421 COVID-19 cases (e.g., 38.3% in South Africa; 15.9% in Morocco; 5.1% in Tunisia; 5.0% in Egypt; 4.5% in Ethiopia), and 65,602 deaths, accounting for 3.4% of global COVID-19 cases for a population that represents 15.8% of the world population (33). Phylogenetic analysis of SARS-CoV-2 performed using 69 sequences from Africa and 155 SARS-CoV-2 sequences from other origins available through GISAID (34), suggested that the SARS-CoV-2 transmitted to Africa were the mutated forms of the Wuhan-Hu-1 strain which first spread to Europe and America and had accumulated mutations. A major characteristic of the African isolates was the high frequency (84.2%) of the A23403G mutation leading to a D614G. Among the African isolates, only the South Africa-2 strain was found to be identical to the Wuhan-Hu-1 strain, while the Benin-3 and Mali-2 isolates were likely to be of Asian origin. More recently, a study (35) investigated 1,414 SARS-CoV-2 genomic sequences collected in the Eastern Mediterranean Region before 12 November 2020 and available through GISAID, including sequences from Saudi Arabia (*n* = 521), the United Arab Emirates (*n* = 186), Oman (*n* = 185), and Egypt (*n* = 150). The other sequences were from viruses isolated in Iran, Iraq, Jordan, Morocco, Palestine, Qatar, and Tunisia. About 65.6% of the viruses belong to clades 20A, 20B, and 20C and non-synonymous mutations leading to two amino acid substitutions, the D614G in the spike and P323L in nsp12, were predominant in most countries. Many country-specific substitutions were also detected. No sequences were available from Djibouti, Afghanistan, Libya, Somalia, Sudan, Syria, and Yemen.

In this context, the situation of Djibouti, located in the horn of Africa with a strategic position at the southern entrance to the Red Sea, where 20,000 ships and 30% of the world's trade passes through (36), is interesting because it hosts the military bases of many countries, leading to a mixing of populations which might favor the circulation of SARS-CoV-2, including particular variants. As early as February 2020, the Republic of Djibouti had worked with the WHO to set up a response plan with rapid implementation to prevent and/or control SARS-CoV-2 spreading in the country (37). The first case of COVID-19 was detected in Djibouti on 17 March 2020, leading the health authorities to apply the strategy aimed at testing any person suspected of being infected with the virus or of being a contact case. Djibouti became the African country that has performed the most SARS-CoV-2 screening in Africa (the country increased its laboratory testing capacity from 100 samples tested by PCR per day up to 2,000 samples per day in April 2020) (14). Between 17 March 2020 and 16 May 2020, Djibouti reported 1,401 confirmed COVID-19 cases including four deaths (a case fatality rate of 0.3%) (13). SARS-CoV-2 continued to spread across the country over the following months. Since the start of the COVID-19 epidemic, the Republic of Djibouti has experienced two epidemic waves. On 18 May 2021, 11,468 confirmed cases of SARS-CoV-2 infection and 152 deaths from severe forms of COVID-19 had been reported in Djibouti (a case fatality rate of 1.32%), according to the Johns Hopkins Resource Center (https://coronavirus.jhu.edu/), with a case fatality rate of 1.10% during the first epidemic wave, 0.36% during the inter-wave period, and 1.61% during the second epidemic wave. This remains lower than the 2.27% fatality reported worldwide.

Although the COVID-19 epidemic has been well-managed with massive screening followed by treatment, the health authorities had no information on the type of strains circulating in the country. The first COVID-19 case in Djibouti was a member of the Spanish special-forces (15, 16). Our study sheds light on the history of COVID-19 in Djibouti. During the first wave of the epidemic, the strains that circulated derived from Wuhan-Hu-1-like strains (also named the “China” strain), namely clade 20A and 20B, but these viruses already carried the D614G found in Europe and USA. The 20A SARS-CoV-2 were genetically related to the Japan/TC-0464/2020 strain and USA/MI-MDHHS-SC20548/2020 strain while the 20B were related to the Somalia/CV1232/2020 and England/ALDP-95C3C7/2020 strain and can be organized in six clusters. It should be noted, the P323L substitution has been frequently observed in nsp12 in the strain spreading in Africa. During the inter-wave phase, the circulating viruses were of the same clade as those present during the first epidemic wave. In contrast, during the second epidemic wave the Wuhan-Hu-1-like viruses were no longer found giving way to variants previously described in Europe (UK variant) and South Africa (South African variant). The UK variant was found in 4% of samples, while the South African variant was found in 96% of samples. South African-like variants circulating in the Djiboutian population during the second epidemic wave belong to clade 20H and were related to viruses circulating in Spain (Spain/CT-HUB00604/2021) and Equatorial Guinea (Equatorial Guinea/85185/2021) Regarding the UK variant found in Djiboutian people, it was genetically related to a strain belonging to clade 20I that circulated the year before in South Korea (South Korea/KDCA830/2020). Very recently (13 June 2021), 52 sequences of SARS-CoV-2 spreading during the second epidemic wave among members of the US department of Defense in Djibouti were deposited in GISAID (GISAID ID Djibouti/NAMRU3-XXXX/2021 where XXXX corresponds to the number of the sequence and 2021 is the year for sample collection). We compared these sequences to the SARS-CoV-2 genomes from Djiboutian' COVID-19 patients (Supplementary Figure 1). Of these viruses sequenced by the US department of Defense in Djibouti, 28 genomes belong to clade 20I (UK variant) and 18 belong to clade 20H (South African variant); the others being 20A and 20B strains. This corroborates our results. However, the frequency of clade 20I was apparently much more widespread among foreigners (American troops) than Djiboutians, while clade 20I was spread in both populations.

In conclusion, the dynamics of SARS-CoV-2 spreading in Djibouti was not so different from that which was observed worldwide, with a first wave dominated by viruses belonging to clades 20A and 20B (Wuhan-Hu-1 -like viruses with viruses presenting several mutations), followed by a decay phase of the epidemic and then a second wave linked to the circulation of variant viruses with a very strong predominance of the South African variant strain of the virus belonging to clade 20H. This second wave turned out to be characterized by an increase in the number of severe forms of COVID-19, leading to more deaths.

## Data Availability Statement

The datasets presented in this study can be found in online repositories. The names of the repository/repositories and accession number(s) can be found in the article/Supplementary Material.

## Ethics Statement

Ethical review and approval was not required for the study on human participants in accordance with the local legislation and institutional requirements. Written informed consent for participation was not required for this study in accordance with the national legislation and the institutional requirements.

## Author Contributions

IO, MHA, DR, and CAD contributed to the design of the study. IAH, ISA, ZAW, and MHA took care of the COVID-19 patients in Djibouti and collected the samples. AL, LB, JD, MB, LH, P-EF, and PC generated and analyzed the virus sequences. IO and CAD prepared the first draft of the manuscript. CAD, MHA, and DR supervised the work. DR obtained the funding for this study. All authors contributed to the article and approved the submitted version.

## Conflict of Interest

The authors declare that the research was conducted in the absence of any commercial or financial relationships that could be construed as a potential conflict of interest.

## Publisher's Note

All claims expressed in this article are solely those of the authors and do not necessarily represent those of their affiliated organizations, or those of the publisher, the editors and the reviewers. Any product that may be evaluated in this article, or claim that may be made by its manufacturer, is not guaranteed or endorsed by the publisher.

## Supplementary Material

Supplementary Figure 1 Phylogenetic tree (obtained using the IQ-TREE software with the GTR Model and 1,000 ultrafast bootstrap 84 repetitions), including SARS-CoV-2 genomes from the COVID-19 Djiboutian patients reported herein (Djibouti/CNSS00XXX-IHU103XXXXXXX/2020 for samples collected in 2020 and Djibouti/CNSS1XXX-IHU103XXXXXXX/2021 for samples collected in 2020; Djibouti/CNSS00XXX: the number XXX can be found in Table 2) compared to the 52 sequences of SARS-CoV-2 spread during the second epidemic wave among members of the US department of Defense in Djibouti (GISAID ID: Djibouti/NAMRU3-XXXX/2021) and several other strains chosen to strengthen the organization of the phylogenetic tree.

Supplementary Table 1 qRT-PCR primers and probes.
