# Supplementary figures and images for: Whole Genome Sequencing of SARS-CoV-2 Strains in COVID-19 Patients From Djibouti Shows Novel Mutations and Clades Replacing Over Time
Source: Front Med (Lausanne). 2021 Sep 1;8:737602. doi: 10.3389/fmed.2021.737602 (PMC8440879; doi:10.3389/fmed.2021.737602)

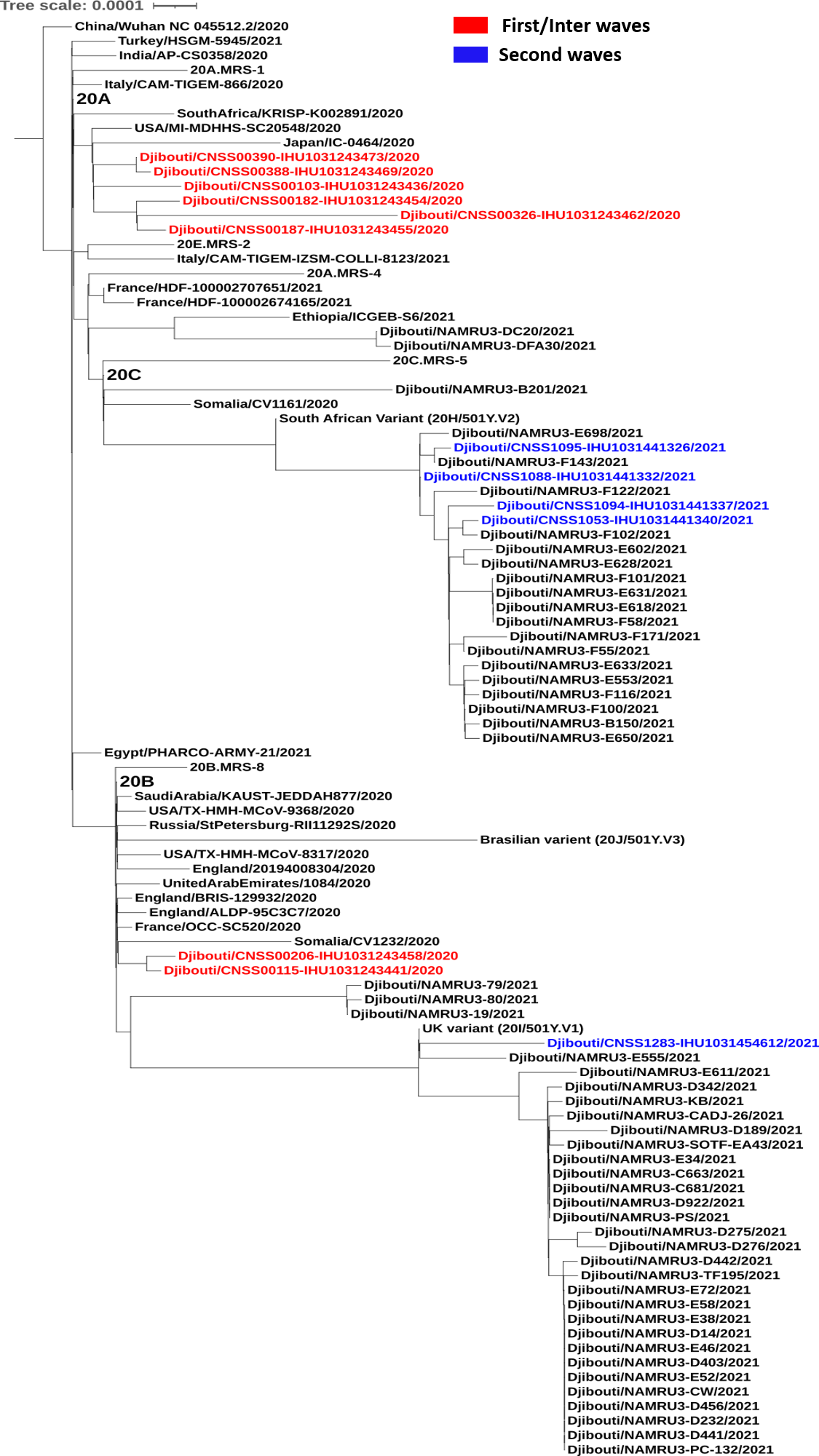

Supplement: Supplementary Figure 1 — Phylogenetic tree (obtained using the IQ-TREE software with the GTR Model and 1,000 ultrafast bootstrap 84 repetitions), including SARS-CoV-2 genomes from the COVID-19 Djiboutian patients reported herein (Djibouti/CNSS00XXX-IHU103XXXXXXX/2020 for samples collected in 2020 and Djibouti/CNSS1XXX-IHU103XXXXXXX/2021 for samples collected in 2020; Djibouti/CNSS00XXX: the number XXX can be found in Table 2) compared to the 52 sequences of SARS-CoV-2 spread during the second epidemic wave among members of the US department of Defense in Djibouti (GISAID ID: Djibouti/NAMRU3-XXXX/2021) and several other strains chosen to strengthen the organization of the phylogenetic tree. [file Image_1.TIF]
