# Supplementary material for: Whole Genome Sequencing of SARS-CoV-2 Strains in COVID-19 Patients From Djibouti Shows Novel Mutations and Clades Replacing Over Time
Source: Front Med (Lausanne). 2021 Sep 1;8:737602. doi: 10.3389/fmed.2021.737602 (PMC8440879; doi:10.3389/fmed.2021.737602)
Supplement: Supplementary Table 1 — qRT-PCR primers and probes. [file Table_1.DOCX]

**Table SI** : qRT-PCR primers and probes

| Identification  of SARS-CoV-2 | SARS-CoV-2 target gene | Primers | Fluorescent dye-tagged  (6-carboxyfluorescein)- probe | Amplification fragment |
| --- | --- | --- | --- | --- |
| Pangenome | E-gene | Sense: 5'-ACAGGTACGTTAATAGTTAATAGCGT-3'  Antisense: 5' ATATTGCAGCAGTACGCACACA-3' | 6FAM-ACACTAGCCATCCTTACTGCGCTTCG | 113 bp |
| Marseille 4 variant  (20A.EU2 (N477S)) | ORF1 | Sense: 5'-GAGGTTTAGAAGAGCTTTTGGTGA-3'  Antisense: 5'- CCAGGTAAGAATGAGTAAACTGGTG-3' | 6FAM-CCTTATTTCATTCACTGTACTCTG | 114 bp |
| UK variant (20I/501Y.V1(N501Y)) | N-gene | Sense: 5'- CATGACGTTCGTGTTGTTTTAGA-3'  Antisense: 5'- CTGAGGGTCCACCAAACGTA-3' | 6FAM-GTCTCTAAATGGACCCCAAAATCA | 110 bp |
| South African variant  (20H/501Y.V2(E484K, N501Y)) | nsp-12 | Sense: 5'- TGAATTGCAGACACCTTTTGA-3'  Antisense: 5'- CAACCCTTGGTTGAATAGTCTTG-3' | 6FAM-TGACATCTTCAATGGGGAATGT | 119 bp |
